# *Akkermansia muciniphila* is a promising probiotic

**DOI:** 10.1111/1751-7915.13410

**Published:** 2019-04-21

**Authors:** Ting Zhang, Qianqian Li, Lei Cheng, Heena Buch, Faming Zhang

**Affiliations:** ^1^ Medical Center for Digestive Diseases the Second Affiliated Hospital of Nanjing Medical University Nanjing 210011 China; ^2^ Key Lab of Holistic Integrative Enterology Nanjing Medical University Nanjing 210011 China; ^3^ Biogas Institute of Ministry of Agriculture and Rural Affairs Chengdu 610041 China; ^4^ Center for Anaerobic Microbial Resources of Sichuan Province Chengdu 610041 China

## Abstract

*Akkermansia muciniphila* (*A. muciniphila*), an intestinal symbiont colonizing in the mucosal layer, is considered to be a promising candidate as probiotics. *A. muciniphila* is known to have an important value in improving the host metabolic functions and immune responses. Moreover, *A. muciniphila* may have a value in modifying cancer treatment. However, most of the current researches focus on the correlation between *A. muciniphila* and diseases, and little is known about the causal relationship between them. Few intervention studies on *A. muciniphila* are limited to animal experiments, and limited studies have explored its safety and efficacy in humans. Therefore, a critical analysis of the current knowledge in *A. muciniphila* will play an important foundation for it to be defined as a new beneficial microbe. This article will review the bacteriological characteristics and safety of *A. muciniphila*, as well as its causal relationship with metabolic disorders, immune diseases and cancer therapy.

## Introduction

Several microbial species are getting increasing attention for their role in modulating the gut microbiota. At present, many diseases and conditions have been reported to be closely related to gut microbiota (de Vos and de Vos, 2012), so it is of great interest to improve the host health by modulating the intestinal bacteria. *Akkermansia muciniphila* (*A. muciniphila*) is a strict anaerobe recently isolated from human faeces and uses the mucin as the sole sources of carbon and nitrogen elements (Derrien *et al*., 2004). This mucin degrader is affected by the nutrients in the mucus layer located at a close distance to the intestinal epithelial (Belkaid and Hand, 2014). Due to this unique function and its high universality and richness in almost all life stages, *A. muciniphila* has opened new avenues for the application in next‐generation therapeutic probiotics (Collado *et al*., 2007; Derrien *et al*., 2008; Belzer and de Vos, 2012; Cani and de Vos, 2017). A series of studies have revealed that *A. muciniphila* regulated metabolic and immune functions, thus protecting mice from high‐fat diets (Derrien *et al*., 2011; Everard *et al*., 2013). Further analysis confirmed *A. muciniphila* can degrade mucin and exert competitive inhibition on other pathogenic bacteria that degrade the mucin (Belzer and de Vos, 2012). These findings provide a rationale for *A. muciniphila* to become a promising probiotic. However, products containing *A. muciniphila* are currently not available worldwide. The exact mechanism underlying *A. muciniphila* interacts with host remains unknown. Based on previous human and animal studies, extensive assessment for *A. muciniphila* is still needed. Here, we will summarize and provide the updated information on the bacteriological characteristics, safety, pathogenicity, antibiotic resistance of *A. muciniphila* and its effects on host health and diseases.

## Characteristics of *A. muciniphila*

**Figure 1:**
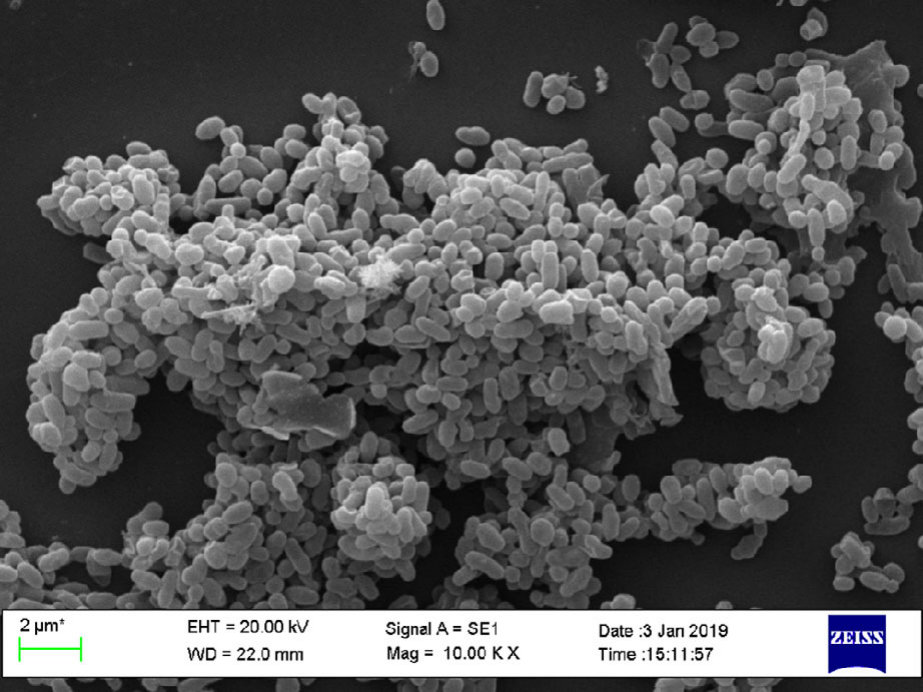
Scanning electronic micrograph of *Akkermansia muciniphila*. The *A. muciniphila* strain was isolated from a healthy Chinese donor for FMT at China fmtBank. Bar represents 2 μm.

*Akkermansia muciniphila* is a bacterium of oval shape, strictly anaerobic, non‐motile and gram‐negative and forms no endospores (Fig. 1). It was historically discovered in 2004 at Wageningen University of the Netherlands when searching for a new mucin‐degrading microbe in human faeces (Derrien *et al*., 2004). *Akkermansia muciniphila* is the first member and the only representative of the phylum *Verrucomicrobia* in the human gut (Miller and Hoskins, 1981; Derrien *et al*., 2010), which is relatively easy to detect (Rajilic‐Stojanovic and de Vos, 2014). The genome of *A. muciniphila* strain MucT (=ATCC BAA‐835T=CIP 107961T) involves one circular chromosome of 2.66 Mbp, which shared a limited number of genes (29%) with its closest relatives in the Verrucomicrobia phylum (van Passel *et al*., 2011). Recently, Guo *et al*. (2017) reported a high genetic diversity of *A. muciniphila* by whole‐genome sequencing, with 5644 unique proteins assembling a flexible open pangenome. They further classified *A. muciniphila* into three species‐level phylogroups, which demonstrated different function features.

It is widely distributed in the intestines of human and animals (Belzer and de Vos, 2012; Lagier *et al*., 2015). *Akkermansia muciniphila* was originally classified as a strictly anaerobic bacterium, but a recent study found that it can tolerate low levels of oxygen, with an oxygen reduction capacity to be 2.26 ± 0.99 mU mg^−1^ total protein (Ouwerkerk, *et al*., (2017b). This property is similar to some intestinal anaerobic colonizers such as *Bacteroides fragilis* and *Bifidobacterium adolescentis*, which could still survive after exposure to ambient air for 48 h. *Akkermansia muciniphila* is abundant in the host intestinal mucosal layer, with a largest number in the caecum. It is found to be ubiquitous in the guts of healthy adults and infants, and accounts for 1–4% of the total gut microbiota starting from early life (Derrien *et al*., 2008).

*Akkermansia muciniphila* is one of the normal gut symbionts throughout our life (Collado *et al*., 2007). This bacterium can stably colonize the human gut within 1 year after birth, and its abundance in the gut eventually reaches the same level as that in healthy adults (Collado *et al*., 2007; Derrien *et al*., 2008), but gradually decreases in the elderly (Collado *et al*., 2007). Previous phylogenetic and metagenomic studies based on hundreds of subjects have found that *A. muciniphila* is one of the top 20 most abundant species detectable in the human gut (Collado *et al*., 2007, 2012; Qin *et al*., 2010; Arumugam *et al*., 2011; Thomas *et al*., 2014; Drell *et al*., 2015). In addition, *A. muciniphila* is reported to be present in human milk (Collado *et al*., 2008). Human milk can act as a carrier for the transfer of *A. muciniphila* from mothers to infants, thereby explaining its presence in the gastrointestinal tract of newborn infants (Collado *et al*., 2007). At this life stage, *A. muciniphila* can successfully colonize the gastrointestinal tract with the active acid resistance system and the ability to degrade human milk oligosaccharides in newborn infants’ stomach (Bosscher *et al*., 2001).

## Culturing *A. muciniphila*

*Akkermansia muciniphila* is divided into three species‐level phylogenetic groups with distinct metabolic features, but current studies still focused on the strain MucT (=ATCC BAA‐835T=CIP 107961T) (Guo *et al*., 2017). *Akkermansia muciniphila* is sensitive to oxygen, and its growth medium is animal‐derived compounds. Therefore, the clinical application of *A. muciniphila* is very limited due to these limitations in culture conditions. Ottman *et al*. (2017a,b) established a genome‐scale metabolic model to evaluate the substrate utilization abilities of *A. muciniphila*. It showed that *A. muciniphila* can utilize the mucin‐derived monosaccharides fucose, galactose and *N*‐acetylglucosamine. These additional mucin‐derived components might be needed for its optimal growth. Plovier *et al*. (2017) reported that *A. muciniphila* can be grown on a synthetic media, in which the mucin is replaced by a combination of glucose, *N*‐acetylglucosamine, peptone and threonine. This synthetic medium is capable of culturing *A. muciniphila* at the same efficiency as the mucin medium, while avoiding all compounds that are incompatible with humans. At the same time, *A. muciniphila* grown on synthetic media was confirmed to be safe for human administration (Plovier *et al*., 2017). A recent study reported that the genome‐scale metabolic model can be used to accurately predict growth of *A. muciniphila* on synthetic media (van der Ark *et al*., 2018). They found that glucosamine‐6‐phosphate (GlcN6P), which exists in the mucin and prompts the adaptation to the mucosal niche, is a necessity for *A. muciniphila*.

Moreover, Ouwerkerk *et al*. (2017a,b) proposed an efficient scalable workflow for the preparation and preservation of viable cells of *A. muciniphila* under strict anaerobic conditions for therapeutic interventions. An anaerobic plating system was used in this process to quantify the recovery and survival of viable cells of *A. muciniphila*. The preserved *A. muciniphila* cells showed very high stability with survival rate of 97.9 ± 4.5% for over 1 year at −80°C in glycerol‐amended medium. These results might pave a way for future clinical studies using *A. muciniphila* as a therapeutic product.

## Safety and pathogenicity of *A. muciniphila*

Currently, a large number of researches on *A. muciniphila* mainly focused on explaining its relationship with diseases, but have not addressed the causality of the bacterium on the diseases (Tables 1 and 2). Several studies focusing on the direct interventions with *A. muciniphila* mostly used animal models (Everard *et al*., 2013; Hanninen *et al*., 2017; Chelakkot *et al*., 2018) (Table 3). Currently, there are no published open clinical trials of *A. muciniphila* for humans and therefore resulting in a lack of strong evidence on the safety of *A. muciniphila* in humans. This could explain why *A. muciniphila* has not been involved in food production or drug use. However, some preliminary studies have indicated this bacterium should be safe for interventions in human. Dubourg *et al*. (2013) reported that even when the abundance of *A. muciniphila* reached a high level of 60% in human following broad‐spectrum antibiotic treatment, no adverse events occurred. Moreover, in an ongoing clinical study, Plovier *et al*. (2017) have first evaluated the safety and tolerability of *A. muciniphila* in overweight subjects. Both live and pasteurized *A. muciniphila* were observed to be tolerated and safe in individuals with excess body weight after 2‐week oral administration of *A. muciniphila*.

**Table 1:** Correlation between *Akkermansia muciniphila* and disease in humans

	Subject	Study type	Study group	Sample type and collection time	Sample detection	Relevance conclusion
Chelakkot *et al*. (2018)	Type 2 diabetes	Observational	–	Faeces, at a selected time point	Metagenome	Compared to patients with type 2 diabetes, healthy human contained more *A. muciniphila* extracellular vesicles (AmEVs) in faeces
Grander *et al*. (2018)	Alcoholic steatohepatitis (ASH)	Observational	ASH: *n* = 21 Non‐obese healthy individuals: *n* = 16	Faeces, at a selected time point	16S rRNA sequencing	Patients with ASH exhibited a decreased abundance of faecal *A. muciniphila* when compared with healthy controls that indirectly correlated with hepatic disease severity. Oral supplementation of *A. muciniphila* promotes intestinal barrier integrity and ameliorates experimental ALD in mice
Dao *et al*. (2016)	Overweight and obese adults	Interventional, limited energy intake for 6 weeks and followed up for 6 weeks	Overweight: *n* = 11 Obesity: *n* = 38	Faeces, T0 = at baseline, T1 = 6 weeks after limiting energy intake, T2 = 12 weeks after stable body weight	Metagenomics, qPCR	Baseline abundance of *A. muciniphila* was negatively correlated with fasting blood glucose, waist‐to‐hip ratio, and subcutaneous fat cell diameter Subjects with high abundance of *A. muciniphila* at baseline had improved insulin sensitivity and other obesity‐related clinical indicators after limiting energy intake
Drell *et al*. (2015)	Children with atopic diseases	Observational	Atopic diseases: *n* = 14 Healthy children: *n* = 15	Faeces, at the age of 5 and 12	Pyrosequencing	A decrease in the abundance of *A. muciniphila* in patients indicated that it plays an important role in IgE‐related atopic diseases compared to healthy people
Brahe *et al*. (2015)	Obese females	Observational	Obese females: *n* = 53	Faeces, at a selected time point	Whole‐genome shotgun sequencing	Abundance of *A. muciniphila* was not associated with insulin resistance and dyslipidaemia
Remely *et al*. (2015a,b)	Overweight adults	Interventional, fasting for 1 week, followed by probiotic intake for 6 weeks	Overweight adults: *n* = 13	Faeces, T1 = before fasting, T2 = during fasting, T3 = 6 weeks after probiotic intervention	qPCR	Compared with that during fasting (T2), the *A. muciniphila* abundance was detected higher before fasting (T1) and after intervention by probiotics (T3)
Remely *et al*. (2015a,b)	Obese individuals	Interventional, 16‐week weight loss diet	Obese individuals: *n* = 33	Faeces, before, during and after the intervention	qPCR	After 16‐week weight loss diet, the abundance of *A. muciniphila* in obese individuals was higher than that before intervention
Kim *et al*. (2014)	Obese females	Interventional, ingestion of Ephedra for 8 weeks, 4 g per day	Obese females:*n* = 7	Faeces, before and after ingestion of Casuarina	16S rRNA sequencing	The increase in *A. muciniphila* abundance was positively correlated with the amount of weight loss in the subjects
Clarke *et al*. (2014)	Outstanding athletes	Observational	High BMI (BMI > 25) Outstanding athlete: *n* = 40 Low BMI (BMI ≤ 25) healthy male: *n* = 23 High BMI (BMI > 25) healthy male: *n* = 23	Faeces, at a selected time point	16S rRNA sequencing	Compared with that in high BMI group, the level of *A. muciniphila* was higher in the group of athletes and healthy men with low BMI values
Escobar *et al*. (2014)	Overweight and obese adults	Observational	Normal weight: *n* = 10 Overweight: *n* = 10 Obesity: *n* = 10	Faeces, at a selected time point	16S rRNA sequencing	The level of *A. muciniphila* had no correlation with BMI value
Zhang *et al*. (2013)	Pre‐diabetes and newly diagnosed type 2 diabetes	Observational	Normal: *n* = 44 Pre‐diabetes: *n* = 64 Type 2 diabetes: *n* = 13	Faeces, at a selected time point	16S rRNA sequencing	*A. muciniphila* abundance was reduced in subjects with pre‐diabetes and type 2 diabetes compared to subjects with normal glucose tolerance
Teixeira *et al*. (2013)	Obese females	Observational	Normal weight: *n* = 17 Obesity: *n* = 50	Faeces, at a selected time point	qPCR	The level of *A. muciniphila* was higher in individuals of normal weight compared to that in obese individuals
Weir *et al*. (2013)	Colorectal cancer	Observational	Colorectal cancer: *n* = 11 Healthy: *n* = 10	Faeces, at a selected time point	16S rRNA sequencing	The level of *A. muciniphila* was elevated in patients with colorectal cancer compared with that in healthy individuals
Candela *et al*. (2012)	Children with atopic diseases	Observational	Atopic diseases: *n* = 19 Healthy children: *n* = 12	Faeces, collected within 3 days	qPCR	The abundance of *A. muciniphila* in children with atopic diseases was missing compared with that in healthy children
Karlsson *et al*. (2012)	Overweight and obese children (4–5 years old)	Observational	Normal weight: *n* = 20 Overweight: *n* = 20 Obesity: *n* = 20	Faeces, at a selected time point	qPCR, T‐RFLP	*A. muciniphila* was less abundant in overweight and obese children than that in normal weight children
Qin *et al*. (2012)	Type 2 diabetes	Observational	Type 2 diabetes: *n* = 71 Healthy controls: *n* = 74	Faeces, at a selected time point	Whole‐genome shotgun sequencing	*A. muciniphila* abundance was higher in faeces of patients with type 2 diabetic compared with that in healthy controls
Collado *et al*. (2012)	Overweight lactating women	Observational	Normal weight: *n* = 34 Overweight: *n* = 22	Breast milk, at 1 month and 6 months after childbirth	qPCR	Compared with that in normal weight women, the abundance of *A. muciniphila* was increased in breast milk of overweight women at 1 month after childbirth
Vigsnaes *et al*. (2012)	UC	Observational	Ulcerative colitis (in active period: *n* = 6, in remission period: *n* = 6) Healthy controls: *n* = 6	Faeces, subjects collected at home	qPCR	Compared with that in healthy controls, the abundance of *A. muciniphila* in faeces of patients with UC was reduced
Wang *et al*. (2011)	Autistic children	Observational	Autistic children: *n* = 23	Faeces, at a selected time point	qPCR	The abundance of *A. muciniphila* was reduced in faeces of autistic children
Swidsinski *et al*. (2011)	Appendicitis, IBD and other diseases	Observational	Appendicitis: *n* = 70 IBD and others: *n* = 400 (100 UC, 100 CD, 50 self‐limiting inflammation, 50 intestinal diverticulum, 50 IBS, 50 health people)	Faeces, at a selected time point	Fluorescence *in situ* hybridization,FISH	The abundance of *A. muciniphila* was inversely proportional to the severity of appendicitis
Collado *et al*. (2010)	Infants (overweight or normal weight pregnant women)	Observational	Infants born to overweight pregnant women: *n* = 26 Infants born to normal weight pregnant women: *n* = 16	Faeces, at 1 month and 6 months	qPCR,FISH‐FCM	Compared with normal weight pregnant women, *A. muciniphila* was more abundant in infants born to overweight pregnant women
Santacruz *et al*. (2010)	Normal weight and overweight pregnant women	Observational	Normal weight pregnant women: *n* = 24 Overweight pregnant women: *n* = 16	Faeces, at a selected time point	qPCR	In normal weight and overweight pregnant women, *A. muciniphila* had no difference in abundance, but its abundance was reduced in obese pregnant women
Png *et al*. (2010)	IBD	Observational	IBD: *n* = 46 (20 of UC, 26 of CD) Healthy people: *n* = 20 (16 of iron deficiency, four of functional abdominal pain)	Tissue specimen (distal colon, proximal colon, terminal ileum), at a selected time point	qPCR	*A. muciniphila* abundance was reduced in IBD patients’ intestinal mucosa compared with in healthy people
Zhang *et al*. (2009)	Morbid obese individuals	Interventional, gastric bypass	Normal weight: *n* = 3 morbid obesity: *n* = 3 After gastric bypass surgery: *n* = 3	Faeces, at a selected time point	16S rRNA sequencing	*A. muciniphila* abundance was reduced in obese individuals compared to normal weight individuals; however, obese individuals received an increased abundance of *A. muciniphila* after gastric bypass
Collado *et al*. (2007)	Healthy human	Observational	1‐month baby: *n* = 50 6 months baby: *n* = 50 12 months baby: *n* = 50 Healthy adults aged 25–35: *n* = 54 Healthy elderly aged 80–82: *n* = 45	Faeces, at a selected time point	qPCR	*A. muciniphila* was colonized in the intestine when a baby was born, and its abundance reached the adult level at the age of 1. With people getting old, the abundance of *A. muciniphila* in the intestine was decreased than before

AmEVs, *A. muciniphila* extracellular vesicles; BMI, body mass index; CD, Crohn's disease; IBD, inflammatory bowel disease; UC, ulcerative colitis.

**Table 2:** Correlation between *A. muciniphila* and disease in animals

	Subject	Study type	Study group	Sample collection	Sample detection	Relevance conclusion
Catry *et al*. (2018)	Nine‐week‐old male C57Bl/6J (WT) and Apoe−/− (KO) mice	Interventional, fed an n‐3 polyunsaturated fatty acid (PUFA)‐depleted (DEF) diet for 12 weeks with or without inulin‐type fructans (ITFs) supplementation for the last 15 days	WT DEF WT DEF ITF KO DEF KO DEF ITF	Caecal content	Illumina Sequencing of the 16S rRNA gene	After prebiotic treatment of inulin‐type fructans, the endothelial dysfunction was improved in mice, and the abundance of *A. muciniphila* was increased
Zhu *et al*. (2017)	Six‐week‐old male C57BL/6J mice	Interventional, treated with fructo‐oligosaccharides and inulin for 6 weeks	Blank control group High dose of FOS group Medium dose of FOS group Low dose of FOS group High dose of inulin group Medium dose of inulin group Low dose of inulin group	Faeces	16S rRNA sequencing	*A. muciniphila* became a dominant species in Verrucomicrobia phylum after treatment with fructo‐oligosaccharides and inulin. It played an important role on maintaining balance between mucin and short‐chain fatty acids
Singh *et al*. (2017)	Male Swiss albino mice	Interventional, HFD (58% fat kcal) for 12 weeks	Normal pellet diet: *n* = 7–8 HFD: *n* = 7–8 Green tea extract: *n* = 7–8 Isomalto‐oligosaccharide: *n* = 7–8 Green tea extract + isomalto‐oligosaccharide: *n* = 7–8	Caecal content	16S rRNA metagenomic sequencing	A combination of green tea extract with isomalto‐oligosaccharide exerted beneficial effects on HFD‐induced alterations in mice and improved *A. muciniphila* abundances
Song *et al*. (2016)	Male C57BL/6J mice	Interventional, HFD plus HPBN of 200 mg/kg for 14 weeks	Low‐fat diet: *n* = 24 High‐fat diet: *n* = 24 High‐fat diet + HPBN: *n* = 24	Faeces	16S rRNA sequencing	Red pitaya betacyanins protect from diet‐induced obesity and its related metabolic disorders, and increase the relative abundance of *A. muciniphila*
Schneeberger *et al*. (2015)	Six‐week male C57BL/6 mice	Interventional, HFD	Normal diet: *n* = 24 High‐fat diet: *n* = 24	Caecal contents, collected at the time mice were sacrificed	qPCR	*A. muciniphila* abundance was reduced in obese mice induced by a high‐fat diet
Gomez‐Gallego *et al*. (2014)	Two‐week BALB/c mice	Interventional	Breastfeeding group: *n* = 12 Infant formula group: *n* = 12 Infant formula group containing intermediate concentration polyamine: *n* = 12 Infant formula group containing high concentration of polyamine: *n* = 12	Oral, stomach, large and small intestine contents	qPCR	Compared with the infant formula group, *A. muciniphila* abundance was increased in the breastfeeding group
Baxter *et al*. (2014)	6–10 weeks male C57BL/6 mice	Interventional, transplanted the faecal bacteria from three colorectal cancer patients and three healthy people to sterile mice (gavage)	Faecal transplantation from healthy adults: *n* = 10 Faecal transplantation from colorectal cancer: *n* = 10 Control group: *n* = 5	Transplanted human and mouse faeces, at day 0 and day 73	16S rRNA sequencing, Illumina sequencing	The abundance of *A. muciniphila* in mice transplanted with faecal bacteria of colorectal cancer patients was higher than that of healthy adults
Hakansson *et al*. (2015)	Wild female C57BL/6 mice	Interventional, 4% DSS feeding for seven consecutive days	Control group not treated with DSS: *n* = 10 Test group treated with DSS: *n* = 10	Colon and caecum contents, at day 7	16S rRNA sequencing, qPCR	The *A. muciniphila* abundance in mice treated with 4% DSS was elevated compared to the untreated group
Zackular *et al*. (2013)	8–12 weeks male C57BL/6 mice	Interventional, tumour‐inducing injection	Control group: *n* = 10 Induced tumour group: *n* = 9	Faeces, collected daily during tumour‐injection	16S rRNA sequencing, qPCR	*A. muciniphila* abundance was elevated in the faeces of tumour mice compared to that in healthy mice
Hansen *et al*. (2012)	NOD mice (non‐obese diabetic mice)	Interventional, 15–21 mice per group, vancomycin (83 mg kg^−1^ day^−1^)	Adult group Newborn rat group Control group	Faeces, collected at the time diagnosed as diabetes or blood glucose > 12 mM	16S rRNA sequencing, pyrosequencing	*A. muciniphila* abundance was decreased in faeces of type 1 diabetic mice, and it was a protective strain of autoimmune diabetes
Berry *et al*. (2012)	6–8 weeks Wt mice and STAT1 ^−/−^ mice	Interventional, the experimental group was given 2% DSS for 7 consecutive days, followed by drinking water for the next 3 days	Experimental group Wt: *n* = 5 STAT1^−/−^: *n* = 5 Control group Wt: *n* = 5 STAT1^−/−^: *n* = 5	Colon and caecum contents, at day 10	16S rRNA sequencing, pyrosequencing	The abundance of *A. muciniphila* in mice treated with 2% DSS was elevated compared to the control group
Sonoyama *et al*. (2010)	Five‐week female BALB/c mice	Interventional, ingesting 4 varieties of rice, then inducing allergic diarrhoea by immunization	(Normal rice): *n* = 6 (Wine rice): *n* = 6 Glutinous rice): *n* = 6 Yukihikari: *n* = 5	Faeces, before immunization	16S rRNA sequencing, qPCR	Compared with other groups, the abundance of *A. muciniphila* in the Yukihikari group was decreased, and the mice in this group were less likely to be induced to develop allergic diarrhoea
Sonoyama *et al*. (2009)	12‐week Syrian hamster	Interventional, dietary intervention for 96 h	Normal diet non‐hibernating mice: *n* = 6 Fasted non‐hibernating mice: *n* = 6 Hibernation mice: *n* = 6	Caecal contents, at the end of the intervention	qPCR	*A. muciniphila* abundance was elevated in the fasted non‐hibernation mice compared to other groups

DSS, dextran sulfate sodium; FOS, fructo‐oligosaccharides; HFD, high‐fat diet; HPBN, hylocereus polyrhizus fruit betacyanins.

**Table 3:** Causal relationship between *A. muciniphila* and disease

	Subject	Study type	Study group	Bacterial intervention	Bacterial status	Sample type	Sample detection	Treatment outcome
Routy *et al*. (2018)	SPF mice	Interventional	αPD‐1: *n* = 5 αPD‐1 + Nacl: *n* = 5 αPD‐1 + *Akkermansia*:* n* = 5 αPD‐1 + *E. hirae*:* n* = 5 αPD‐1 + *Akkermansia* & *E. hirae*:* n* = 5 αPD‐1 + Alistipes ind: *n* = 5	Mice exhibiting non‐response FMT‐induced dysbiosis were compensated with *A. muciniphila* alone or combined with *E. hirae* or control bacteria during PD‐1 mAb‐based therapy	Viable	Faeces	Metagenomic analysis	FMT from cancer patients who did not respond to ICIs into germ‐free or antibiotic‐treated mice failed to ameliorate the antitumour effects of PD‐1 blockade. Oral supplementation with *A. muciniphila* after FMT with non‐responder faeces restored the efficacy of PD‐1 blockade
Chelakkot *et al*. (2018)	Male 6–8 week C57BL/6 mice	Interventional	ND: *n* = 5–7 HFD: *n* = 5–7 ND with AmEVs: *n* = 5–7 HFD with AmEVs: *n* = 5–7	Orally administered with 10 μg AmEVs once every two days for two weeks	Viable	Faeces, colon tissue, rat tail vein blood	16S rRNA sequencing, immunohistochemistry, immunoblotting	*A. muciniphila* extracellular vesicles may improve metabolic function by altering intestinal permeability and barrier integrity in high‐fat diet mice
Plovier *et al*. (2017)	10‐ to 11‐week‐old male C57BL/6J mice; Human subjects with excess body weight	Interventional	Mice: ND HFD HFD live Akk mucin HFD live Akk synthetic HFD pasteurized AKK HFD Amuc_1100× Human: Placebo Akk Synthetic – 10^10^ Akk Synthetic – 10^9^ Akk Pasteurized – 10^10^	Human subjects were assigned to receive either a daily dose of placebo (an equivalent volume of sterile PBS containing glycerol), 10^10^ CFU live *A. muciniphila* (Akk S – 10^10^), 10^9^ CFU live *A. muciniphila* (Akk S – 10^9^), or 10^10^ CFU pasteurized *A. muciniphila* (Akk P – 10^10^) for 3 months	Live and pasteurized	Intestinal tissue, blood	Real‐time qPCR	*A. muciniphila* retains its efficacy when grown on a synthetic medium. Pasteurization of *A. muciniphila* enhanced its capacity to reduce fat mass development, insulin resistance and dyslipidaemia in mice. Administration of live or pasteurized *A. muciniphila* grown on the synthetic medium is safe in humans
Hanninen *et al*. (2017)	Non‐obese diabetic mice	Interventional	Microbiota transplantation group *A. muciniphila* group Control group	(i) 330 μl bacterial suspension from mice with low diabetes incidence rate, twice daily for three consecutive days (ii) Orally administered 2 × 10^8^ cfu *A. muciniphila*, three times a week for 7 weeks (iii) Orally administered 2 × 10^6^ cfu *A. muciniphila*, three times a week for 7 weeks	Viable	Faeces, caecal and colon contents	16S rRNA sequencing	Transplanting the gut microbiota of mice with low diabetes incidence to mice with high diabetes incidence did not reduce the morbidity of diabetes; but transplanting the single strain *A. muciniphila* to mice with high incidence of diabetes can reduce the morbidity of diabetes
Li *et al*. (2016)	Eight‐week‐old male Apoe−/− mice	Interventional	NCD: *n* = 8–10 WD: *n* = 8–10 WD+Akk: *n* = 8–10 WD+hk‐Akk: *n* = 8–10 WD+PBS: *n* = 8–10	The Western diet‐fed mice were further separated into three groups: a group receiving daily oral gavage with live *A muciniphila* (WD+Akk), a group receiving daily oral gavage with heat‐killed *A muciniphila* (WD+hk‐Akk), and a third gavaged with PBS as vehicle control (WD+PBS)	Live	Aorta and ileum	Real‐time qPCR	Oral gavage with *A muciniphila* protected against western diet‐induced atherosclerotic lesion formation in Apoe^−/−^ Mice
Shin *et al*. (2014)	C57BL/6 mice	Interventional	NCD‐fed control mice: *n* = 6 HFD‐fed control mice: *n* = 6 HFD‐fed metformin‐treated mice: *n* = 6 HFD‐fed Akk‐administered mice: *n* = 6	The bacteria were harvested at the late exponential growth phase, suspended in thioglycolate–phosphate‐buffered saline (PBS) (4.0 × 10^8^ cfu) and orally administered to HFD‐fed mice (HFD‐Akk; *n* = 6)	Viable	Faeces	16S rRNA gene sequences with 454 pyrosequencing	Oral administration of *Akkermansia muciniphila* to HFD‐fed mice without metformin significantly enhanced glucose tolerance and attenuated adipose tissue inflammation by inducing Foxp3 regulatory T cells (Tregs) in the visceral adipose tissue
Kang *et al*. (2013)	Specific pathogen free C57BL/6 mice	Interventional	Water: *n* = 5 2% DSS: *n* = 5 2% DSS + *A. muciniphila*:* n* = 5 2% DSS + AmEV: *n* = 5	2% DSS was administered to female C57BL/6 mice for 5 days, and then, mice were treated with 2% DSS and *A. muciniphila* (5 × 108 CFU per mouse), and treated with 2% DSS and *A. muciniphila*‐derived EV (AmEV, 100 mg/mouse).	Viable	Small intestinal fluids and stools	Metagenome sequencing	*A. muciniphila*‐derived extracellular vesicles have protective effects in the development of DSS‐induced colitis
Everard *et al*. (2013)	10‐week C57BL/6 mice	Interventional	CT control diet group: *n* = 4 HF high‐fat diet group (60% fat): *n* = 6 HF‐AKK group (+ *A. muciniphila* live bacteria): *n* = 5 HF‐K‐AKK group (+ *A. muciniphila* heat‐killed bacteria): *n* = 5	Intragastric administration of *A. muciniphila* (live bacteria, heat‐killed bacteria, 2 × 10^8^ cfu 0.2 ml^−1^)	Live and heat‐killed	Caecal contents, collected every day	16S rRNA sequencing, qPCR	*A. muciniphila* abundance was decreased in mice with diabetes and obesity caused by high‐fat diet, and the metabolic function of mice could be improved by intragastric administration of live *A. muciniphila*

AmEVs, *A. muciniphila* extracellular vesicles; DSS, dextran sulphate sodium; FMT, faecal microbiota transplantation; HFD, high‐fat diet; ICIs, immune checkpoint inhibitors; NCD, normal chow diet; ND, normal diet; PBS, phosphate‐buffered saline; SPF, specific pathogen‐free; WD, Western diet.

As for the pathogenicity of *A. muciniphila*, it has not yet been clearly associated with any disease or sign of illness (Derrien *et al*., 2010). The potential pathogenicity of *A. muciniphila* was mainly due to its process from adhesion to degradation of the intestinal mucus layer, which may involve some initial pathogenic behaviours (Donohue and Salminen, 1996; Tuomola *et al*., 2001; Derrien *et al*., 2010). Unlike pathogens, *A. muciniphila* as a mucin‐degrading agent mainly stays in the outer mucosal layer and does not reach the inner mucosal layer, but bacteria reaching the inner layer have been shown to be required for pathogenicity (Gomez‐Gallego *et al*., 2016). Although degrading mucin itself is a pathogen‐like behaviour (Donohue and Salminen, 1996), it is considered a normal process in the intestinal self‐renewal balance (Gomez‐Gallego *et al*., 2016). Moreover, it is reported that *A. muciniphila* may maintain host intestinal microbial balance by converting mucin into beneficial by‐products (Derrien *et al*., 2008). To date, there is no evidence that *A. muciniphila* alone causes pathogenicity; nevertheless, it is not known whether it may cause diseases in synergy with other bacteria.

*Akkermansia muciniphila*, as a gram‐negative bacterium, contains lipopolysaccharide, but it is not associated with endotoxemia. This bacterium even reduced the endotoxin level associated with high‐fat diets in mice (Everard *et al*., 2013). Mucin degradants are known to regulate host immune system through signals such as tumour necrosis factor alpha (TNF‐α), interferon gamma (INF‐γ), interleukin‐10 (IL‐10) and IL‐4 (Derrien *et al*., 2011; Collado *et al*., 2012; Andersson *et al*., 2013). There was evidence that a decreased level of the anti‐inflammatory cytokines IL‐10 and IL‐4 and an elevated level of pro‐inflammatory cytokines TNF‐α and IFN‐γ were associated with an increased level of *A. muciniphila* (Collado *et al*., 2012). From a genetic point of view, colonization of *A. muciniphila* in sterile mice did not cause side‐effects or the upregulated expression of pro‐inflammatory cytokines (Derrien *et al*., 2011). Intestinal anti‐inflammatory and protective effects were thought to be closely related to *A. muciniphila* (Png *et al*., 2010; Candela *et al*., 2012). Hence, we suggest that treatment with *A. muciniphila* should be safe with a rationale.

## Colonization of *A. muciniphila* and its interaction with the host

The ability of *A. muciniphila* to adhere to the mucus layer was considered to be a beneficial probiotic characteristic (Derrien *et al*., 2010; Everard *et al*., 2013; Chelakkot *et al*., 2018; Hanninen *et al*., 2018). The intestinal mucosal layer mainly protects epithelial cells from microbial attacks and provides growth energy for microorganisms that use it as a nutrient. A low level of *A. muciniphila* in the intestine may result in the thinning of the mucosa, thus leading to a weakening of the intestinal barrier function, and making it easier for the toxins to invade the host. The relationship between *A. muciniphila* and the host is not only reflected in the intake, utilization and consumption of energy associated with glucose, protein and lipid metabolism, but also in the integrity of mucosal layer and related mucosal immune response. *Akkermansia muciniphila* not only participates in the host immune regulation, but also enhances the integrity of the intestinal epithelial cells and the thickness of the mucus layer, thereby promoting intestinal health (Everard *et al*., 2013; Reunanen *et al*., 2015).

Microorganisms on the surface of the intestinal mucosa are known to contribute more to host immunity, and *A. muciniphila* is a typical representative (Nieuwdorp *et al*., 2014). The host's nutrient environment could affect the growth of *A. muciniphila* in the intestine. For example, the property of *A. muciniphila* degrading mucin can be defined as a competitive advantage when the host is in nutritional deficiencies such as during fasting and in malnutrition. This was confirmed by the experiment on hamsters that the abundance of *A. muciniphila* was significantly increased after fasting (Sonoyama *et al*., 2009). The level of mucin in the intestine of rats fed with arabinose or inulin was significantly increased, and this change also contributed to the abundance of *A. muciniphila*.

In turn, the host will also benefit from the colonization of *A. muciniphila*. *A. muciniphila* was colonized in the sparse mucus layer, and it therefore was closer to the intestinal epithelial cells than other microorganisms colonized in the intestinal lumen. Its metabolites, such as propionic acid, were also present in the mucus layer close to the intestinal epithelial cells and were easily accessible to the host. Propionic acid can act on the host through Gpr43 (G protein‐coupled receptor 43), while other short‐chain fatty acids through Gpr41, thus causing a series of downstream pathway changes to achieve immunomodulatory effects (Le Poul *et al*., 2003; Maslowski *et al*., 2009).

*In vivo*,* A. muciniphila* was colonized in sterile mice and the effective colonization was highest in the caecum (Derrien *et al*., 2011). This may be explained by the reason that most of the mucin was produced in the caecum. The whole transcriptome analysis of intestinal tissue samples indicated that *A. muciniphila* regulated the expression of approximately 750 genes, with the changes mainly focused on genes associated with immune responses. *In vitro*, propionic acid and butyric acid are the main metabolites of *A. muciniphila*. *A. muciniphila* regulated the expression of 1005 genes in intestinal tissue, of which 503 genes were upregulated and 502 genes were down‐regulated. While *Faecalibacterium prausnitzii* only affected the expression of 190 genes, of which 86 were upregulated, and 104 genes were downregulated (Lukovac *et al*., 2014). Consequently, *A. muciniphila* can regulate the host's metabolism and immune function. However, the causal relationship between the microbes and host genomes is very complicated and needs to be further evaluated (Wang *et al*., 2018a,b).

## *Akkermansia muciniphila* regulated the balance between health and disease

*Akkermansia muciniphila* has recently been considered as a significant factor in human physiology, including homeostatic and pathological conditions. A large number of human and animal studies have addressed the associations between the abundance of *A. muciniphila* and various disorders and diseases (Tables 1 and 2). The decreased level of *A. muciniphila* is considered to be related to the development of some diseases. Amongst which, the majority were metabolic disorders and inflammatory diseases, including obesity, type 2 diabetes, inflammatory bowel disease (IBD), autism and atopy. However, Weir *et al*. (2013) found that the level of *A. muciniphila* was obviously elevated in patients with colorectal cancer compared with that in healthy individuals. This negative correlation might be associated with some confounders such as diet and medication. For example, food intake was greatly reduced in patients with colorectal cancer, while fasting is reported to be involved in increasing the level of *A. muciniphila* (Remely *et al*., 2015a,b). A small sample size of patients might be another influencing factor. Moreover, some studies showed that no relation with *A. muciniphila*‐like bacteria was observed by metagenomic analysis (Zeller *et al*., 2014; Yu *et al*., 2017).

Recently, the research models of microbiome are facing a shift from focusing on association with a causality in recent years. For example, the beneficial therapeutic effects can be observed when the bacteria were administered in a viable form (Table 3). Consequently, *A. muciniphila* may become a biomarker of host health status, indicating the state of disease progression (Png *et al*., 2010; Swidsinski *et al*., 2011; Berry and Reinisch, 2013).

Unexpectedly, a recent study showed that pasteurized *A. muciniphila* can also prevent obesity and related complications, with the effectiveness be even better than live bacteria (Plovier *et al*., 2017). Even more exciting, the research team purified the outer membrane protein of *A. muciniphila*, Amuc_1100, which may exert this beneficial effect. Amuc_1100 was stable during pasteurization and interacted with Toll‐like receptor 2 to improve intestinal barrier function and to perform part of the probiotic function alone. Consistent with this finding, Ottman *et al*. (2017a,b) also found that Amuc_1100 could activate TLR2 and TLR4 to increase IL‐10 production and thus regulating immune response and intestinal barrier function This finding is significant and provides an important theoretical basis for the application of *A. muciniphila* in clinical treatments. However, the proved activity of *A. muciniphila* in pasteurized form has caused another controversial problem. The use of the term probiotic, which was specifically defined as live microorganisms by the Expert Panel from the Food and Agriculture Organization of the United Nations in 2001, may be misleading. A recent review stated that probiotic applications can be either live or dead forms (Hai, 2015). Regarding this modified definition, the Expert Panel previously declared that a dead probiotic is not approved. They demonstrated that if dead organisms have beneficial properties, they should be defined as a different term instead of probiotic. The perfect definition of probiotics needs further improvement in future.

### Metabolic disorders and *A. muciniphila*

*Akkermansia muciniphila* is abundant in the gut microbiota of healthy individuals and exerts the effect of preventing and treating obesity, type 2 diabetes and other metabolic dysfunctions (Png *et al*., 2010, Santacruz *et al*., 2010; Karlsson *et al*., 2012; Everard *et al*., 2013; Zhang *et al*., 2013). Previous studies found that its abundance was inversely proportional to the body weight of mice and humans (Derrien *et al*., 2010; Santacruz *et al*., 2010; Karlsson *et al*., 2012; Everard *et al*., 2013; Teixeira *et al*., 2013). *Akkermansia muciniphila* can significantly increase glucose tolerance and attenuate adipose inflammation in obese mice by inducing Foxp3 regulatory T cells (Shin *et al*., 2014). With the application of probiotics to overweight subjects after fasting, an obviously increased level of *A. muciniphila* was observed (Remely *et al*., 2015a,b). Moreover, an interventional study with *Akkermansia* showed that the level of blood lipopolysaccharide, which functioned as an indicator of gut permeability, was significantly decreased in obese mice after the administration of *Akkermansia* (Everard *et al*., 2013). Similarly, another study established that *Akkermansia*‐derived extracellular vesicles could regulate the intestinal permeability and barrier integrity and thus affect the metabolic functions in mice with a high‐fat diet (Chelakkot *et al*., 2018). Dao *et al*. (2016) reported that the baseline level of *A. muciniphila* in obese patients was negatively related to the fasting blood glucose, waist‐to‐hip ratio and subcutaneous fat cell diameter. And after limiting energy intake for 6 weeks, patients with a high abundance of *A. muciniphila* at baseline had significantly improved insulin sensitivity and other obesity‐related clinical indicators. *Akkermansia muciniphila* can be therefore used as a metabolic marker to indicate the reduction in the risk of obesity (Brahe *et al*., 2015), and it might be directly used to improve the glucose and lipid metabolism to treat obesity.

Recently, Chelakkot *et al*. (2018) reported that compared to patients with type 2 diabetes, healthy human contained more *A. muciniphila* extracellular vesicles (AmEVs) in faeces. Another study found that the abundance of *A. muciniphila* was reduced in subjects with pre‐diabetes and type 2 diabetes compared to subjects with normal glucose tolerance (Zhang *et al*., 2013). The relationship between *A. muciniphila* and type 2 diabetes was also reflected in cases using metformin (Lee and Ko, 2014). High levels of *A. muciniphila* in patients seemed to contribute to enhancing the efficacy of metformin (Shin *et al*., 2014). This was confirmed by the correlation between an increased *A. muciniphila* level and the effectiveness of metformin in a recent study (Forslund *et al*., 2015). Although the mechanisms involved are not fully understood (van Passel *et al*., 2011; Swidsinski *et al*., 2011; Everard *et al*., 2013; Cani and Everard, 2014; Shin *et al*., 2014), these animal experiments and related human studies have provided strong support for *A. muciniphila* in regulating energy homeostasis and glucose metabolism.

Several animal experiments and one human study have used *A. muciniphila* for direct intervention to evaluate its effectiveness in treating metabolic diseases. Initially in 2013 (Everard *et al*., 2013), Everard *et al*. reported that the abundance of *A. muciniphila* was decreased in mice with diabetes and obesity caused by high‐fat diet, and the metabolic function of mice could be improved by intragastric administration of viable *A. muciniphila*. In 2017, Hanninen *et al*. (2017) established that transplanting the gut microbiota of mice with low incidence of diabetes, into the mice with high incidence of diabetes, did not reduce the morbidity of diabetes, but transplanting the single strain *A. muciniphila* into the mice with high incidence of diabetes can reduce the morbidity of diabetes. Chelakkot *et al*. (2018) reported that the intervention of oral administration with AmEVs may improve metabolic function by altering intestinal permeability and barrier integrity in high‐fat diet mice. Thus, based on these direct interventional studies, *A. muciniphila* could be a very promising beneficial microbe for treating metabolic disorders. Most importantly, Plovier *et al*. (2017) have implemented a clinical study to evaluate the efficacy of *A. muciniphila* on metabolic syndrome. Currently, complete results have not been published, but the preliminary human data at least suggested that oral administration of this bacterium is safe. Altogether, these results demonstrate that *A. muciniphila* promises to be a potential therapy to treat metabolic diseases.

### Immune diseases and *A. muciniphila*

A decreased abundance of *A. muciniphila* in children with atopic diseases indicated that it plays an important role in IgE‐related atopic diseases (Drell *et al*., 2015). The correlation between a low level of *A. muciniphila* and immune response in atopic children suggested that *A. muciniphila* could interact with intestinal epithelial cells to produce IL‐8 for immunomodulatory effects (Drell *et al*., 2015; Reunanen *et al*., 2015). In addition, the reduction in the number of *A. muciniphila* was closely related to the occurrence of IBD (Png *et al*., 2010; Rajilic‐Stojanovic *et al*., 2013). The abundance of *A. muciniphila* was significantly decreased in the intestinal mucosa of IBD patients compared to that in healthy people (Png *et al*., 2010). Kang *et al*. (2013) recently found that AmEVs could regulate intestinal immunity and homeostasis and exert protective effects in the development of dextran sulfate sodium‐induced colitis in mice. However, there is still a lack of human experiments that directly interfere with *A. muciniphila* to illustrate the causal relationship between this microbe and host immune diseases.

### Cancer therapy and *A. muciniphila*

Recently, three consecutive articles published in 2018 have shown the importance of gut microbiota combined with anti‐PD‐1 antibody in cancer therapy (Gopalakrishnan *et al*., 2018; Matson *et al*., 2018; Routy *et al*., 2018). Routy *et al*. (2018) analysed the relationship between the therapeutic efficacy of immune checkpoint inhibitors and the gut microbiota in patients with different cancers. They found that the intestinal level of *A. muciniphila* was significantly increased in patients with a positive response to the immune checkpoint inhibitor PD‐1 antibody. Furthermore, when the faecal microbiota from patients who responded positively to the immunotherapy were transplanted to sterile mouse, the corresponding positive response to the anti‐PD‐1 antibody was achieved. But when the faecal bacteria from patients who did not respond to the immunotherapy were transplanted to sterile mice, the native response was observed. Excitingly, the mice could recover their response to the anti‐PD‐1 antibody after oral administration of *A. muciniphila*. In addition, Matson *et al*. (2018) reported *A. muciniphila* abundance was observed in four metastatic melanoma patients with clinical response to anti‐PD‐1‐based immunotherapy. After gavaged with faecal material from responding patient donors, improved tumour control and better efficacy of immunotherapy was observed in a mouse melanoma model. Gopalakrishnan *et al*. (2018) also found a higher level of good intestinal bacteria in the melanoma patients who responded to the treatment of PD‐1 blockade. Combining three studies, Gharaibeh *et al*. (Gharaibeh and Jobin, 2018) concluded that there was a signal for more *A. muciniphila* in responders. The above results indicate that cancer immunotherapy combined with *A. muciniphila* as one of important probiotics in selective microbiota transplantation (Wu *et al.,* 2019) is expected to achieve better clinical results for patients in the near future.

Consistently, Wang *et al*. (2018a,b) reported one patient with high‐grade metastatic urothelial carcinoma showed immune checkpoint inhibitors (ICI)‐associated colitis after a trial of combined CTLA‐4 and PD‐1 blockade. After ICI‐associated colitis in the patient was successfully treated with FMT, donor‐derived bacteria were observed to be effectively colonized in the patient's intestinal tract, with an obviously higher level of *A. muciniphila*. Consequently, *A. muciniphila* has shown its potential role in the treatment of cancer, and this role needs to be further confirmed by researchers.

## Conclusions

*Akkermansia muciniphila*, as a potential probiotic that can make good use of gastrointestinal mucin, is inextricably linked to host metabolism and immune response. It promises to be a therapeutic target in the microbiota‐related diseases, such as colitis, metabolic syndrome, immune diseases and cancer. Preliminary human data suggest oral administration of *A. muciniphila* is safe, but its effect needs to be further verified in more human clinical trials in the near future.

## Conflict of interest

None declared.
